# The Impact of Polymerized Whey Protein on the Microstructure, Probiotic Survivability, and Sensory Properties of Hemp Extract-Infused Goat Milk Yogurt

**DOI:** 10.3390/foods14010066

**Published:** 2024-12-29

**Authors:** Hao Shi, Kalev Freeman, Eric Kawka, Monique McHenry, Mingruo Guo

**Affiliations:** 1 Department of Nutrition and Food Sciences, The University of Vermont, Burlington, VT 05405, USA; Hao.Shi@uvm.edu; 2 Departments of Emergency Medicine and Pharmacology, The University of Vermont, Burlington, VT 05405, USA; Kalev.Freeman@uvm.edu; 3 Cattis Scientific, Hardwick, VT 05843, USA; eric@cattisvt.com; 4 Department of Pharmacology and Plant Biology, The University of Vermont, Burlington, VT 05405, USA; mmchenry@uvm.edu

**Keywords:** goat milk yogurt, polymerized whey protein, hemp extract, flavor, probiotics

## Abstract

Goat milk yogurt infused with hemp extract (HE) is a novel dairy product; however, the unpleasant flavors from hemp terpenes and goat milk may impact its acceptance and popularity. This study aimed to investigate the effect of polymerized whey protein (PWP) on mitigating the hempy flavor of HE-infused goat milk yogurt and its impact on the physicochemical properties, microstructure, and probiotic survivability. Goat milk yogurt samples were infused with either nothing (plain flavor), HE, HE plus whey protein isolate, or HE plus PWP. Compared with plain goat milk yogurt, the addition of PWP in HE goat milk yogurt greatly improved the viscosity. The sensory evaluation results (N = 19) indicated that PWP significantly improved the consistency and decreased the hempy flavor of HE goat milk yogurt, although there was no difference in consumer acceptance. The microstructure analysis revealed that adding PWP formed a compact gel network compared to the irregular open protein matrixes in other groups. In conclusion, PWP not only improved the consistency of goat milk yogurt but was also useful in mitigating the hempy flavors of HE-infused goat milk.

## 1. Introduction

Goat milk and its products increase in popularity year after year because of consumers’ appreciation and particular nutrition demands [1]. Compared to cow milk, goat milk has smaller fat globules, a higher proportion of short- and medium-chain fatty acids, zinc, iron, and magnesium, and also exhibits antibacterial characteristics [2]. In addition, due to its unique physicochemical properties and protein compositions [3], goat milk is easier to digest and less allergenic, enabling it as a bovine milk alternative for people with gastrointestinal disorders and special dietary demands [4].

Besides its unique nutritional properties, goat milk is a suitable vehicle to deliver probiotics [5]. Probiotics, mostly live bacteria, exert promising health benefits, such as maintaining a healthy microbiome (e.g., alleviating constipation and diarrhea), managing type 2 diabetes, and improving cardiovascular health [6,7,8,9]. The divergent probiotic groups of *Bifidobacterium* and *Lactobacillus* considerably benefit health in various ways. For example, they can detoxify environmental pollutants and food toxins, synthesize micronutrients, ferment indigestible fiber in the colon, suppress the growth of harmful bacteria, attenuate the immune system, and modulate metabolic activities [10,11,12]. The overall health benefits of goat milk and probiotics provide a rationale for developing functional probiotic goat milk yogurt. However, studies indicated that goat milk yogurt showed lower consumer acceptability than cow milk yogurt, even with the addition of fruit pulps [13,14]. This deficiency may be attributed to its flavor and texture. The majority of flavor compounds in goat milk yogurt are short- and medium-chain fatty acids, such as the caproic (C6), caprylic (C8), and capric (C10), contributing to the distinctive flavor of goat milk products [15]. Moreover, the lower level of α_S1_-casein in goat milk yogurt results in a weaker consistency than cow milk yogurt [16]. Therefore, the flavor and texture properties should be considered to make goat milk yogurt more acceptable.

Industrial hemp is a valuable agricultural crop. Hemp products are blooming in the US, such as hemp seed milk, hemp seed protein, flour, and paper/clothes made of hemp fibers [17]. However, studies on hemp extract-infused dairy products are limited, creating an excellent research opportunity to develop goat milk yogurt with hemp extract. Industrial hemp, belonging to *Cannabis sativa*, is cultivated with less than 0.3% of the psychoactive compound delta-9 tetrahydrocannabinol (THC) [18]. Hemp extract (HE) oil is abundant whichn cannabinoids (mainly cannabidiol, [CBD]), terpenes, and flavonoidswhichch exhibit promising health benefits, e.g., anti-cancer and anti-tumorigenic activities, anti-inflammatory properties, alleviating adverse effects from chemotherapy, and improving stress-related insomnia [19,20,21,22,23]. Hemp extract-infused products may be considered functional foods [24]; however, the characteristic astringent flavor and taste of hemp extracts (namely due to hemp terpenes) may affect their acceptance among consumers [25]. Several studies have described the flavor properties of hemp products. The addition of hemp flour at only 20% can cause a reduction in flavor acceptance of the bread compared to the control gluten-free starch bread [26]. Later, Hayward and McSweeney [27] also explored the acceptability of bread made with hemp (*Cannabis sativa* subsp. sativa), indicating a bitter and astringent taste of bread from hemp flour. Teterycz et al. [28] developed a hempseed (*Cannabis sativa* L.) enriched pasta and reported a bitter taste from their sensory evaluation test.

Therefore, to improve the potential deficiencies of HE-infused goat milk yogurt, a novel manufacturing process using polymerized whey protein (PWP) to encapsulate flavor compounds may be a useful method to improve the texture and also mitigate the flavor of HE-infused goat milk yogurt. Prior work has shown that flavor compounds can be reduced by the fermentation via lactic acid bacteria [29], trapped by additives (e.g., cyclodextrin), and masked by flavoring agents [30]. In addition, increasing the content of total solids and/or adding stabilizers in milk have also been widely used in the dairy manufacturing process to improve texture and consistency [31,32]. PWP is a soluble protein aggregate formed under controlled temperature and protein concentration through thermal treatment. Compared with the native whey protein, PWP exhibits improved functional properties, such as gelation and film-forming properties, and has been used in the food industry as a thickening agent, stabilizer, and wall material of microencapsulation [16]. It has been reported that PWP not only improved the physical properties of yogurt and yogurt-like products but also mitigated unpleasant flavors of goat milk products [33,34,35]. β-Lactoglobulin (β-LG), a dominating protein of whey proteins, is a member of the lipocalin family that is capable of binding small ligands, such as retinol, vitamins, and fatty acids [36,37]. When heated, β-LG unfolds, resulting in the extensive exposure of hydrophobic groups and reactive nucleophilic sites (–SH and charged animo acid residues). It may be capable of binding with short/medium-chain fatty acids and aromatic compounds [38]. Based on this unique functional property of PWP, we reasoned that PWP may improve the texture and flavor properties of HE-infused goat milk yogurt.

The objectives of this study were (1) to develop hemp extract (HE)-infused probiotic goat milk yogurt and (2) to investigate the effect of PWP on mitigating the hempy and goaty flavor in HE-infused goat milk yogurt.

## 2. Materials and Methods

### 2.1. Materials

Goat milk (Oak Knoll Dairy, Windsor, VT, USA), whole cow milk (Monument Dairy, Weybridge, VT, USA), and sugar (Domino^®^, New York, NY, USA) were purchased from the local grocery store. Whey protein isolate was bought from Fonterra, Auckland, New Zealand. Full spectrum hemp extract was provided by Cattis Scientific, Hardwick, VT, USA. Starter cultures (ABY-3) were provided by Chr. Hansen, Milwaukee, WI, USA, consisting of *Streptococcus thermophilus*, *Lactobacillus delbrueckii* subsp. *bulgaricus*, *Bifidobacterium* BB-12, *Lactobacillus acidophilus* LA-5. All chemicals and reagents used in this study were analytical grade and purchased from Fisher Scientific (Hampton, NH, USA).

### 2.2. Methods

#### 2.2.1. Preparation of Polymerized Whey Protein

Polymerized whey protein (PWP) was prepared according to the method of Wang et al. [31]. Whey protein isolate solution (10%, *w*/*v*) was prepared at room temperature and fully hydrated at 4 °C overnight. The next day, it was warmed up to room temperature, and the pH was adjusted to 7.5 using 2 M sodium hydroxide. The whey protein solution was then heated at 85 °C for 30 min and rapidly cooled down using cold running water.

#### 2.2.2. Yogurt Manufacturing

**Figure 1:**
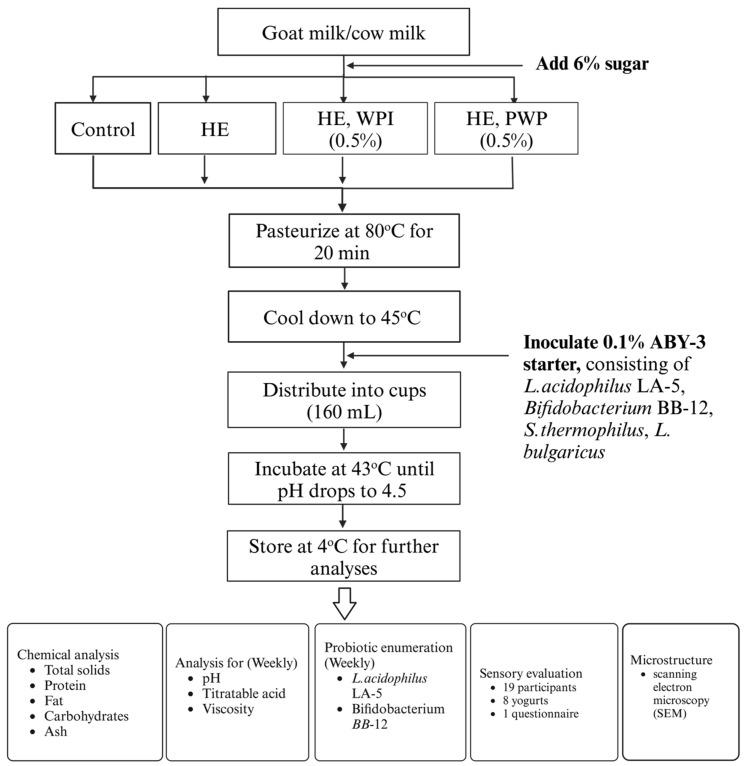
Flow chart of yogurt samples preparation.

Raw hemp extract was decarboxylated by heating at 155 °C for 2 h. During the decarboxylation process, the acid form of cannabinoids was converted to the neutral form. Four different goat milk yogurts were infused with either nothing (control, plain flavor), hemp extract (HE), HE with unheated whey protein isolate solution (WPI, 0.5%, *v*/*v*), and HE with PWP (0.5%, *v*/*v*); see Figure 1. All the yogurts fortified with HE were designed to contain 25 mg CBD per serving (160 mL). Decarboxylated HE oil was melted in hot water and then emulsified with WPI or PWP using a hand-held blender at high speed. Goat milk was mixed with sugar (6%, *w*/*v*) and fortified with or without HE, WPI, and PWP based on the above experimental design. Then, the goat milk was pasteurized in a water bath at 80 °C for 20 min and cooled down to 45 °C before inoculating with ABY-3 starter cultures (0.1%, *w*/*v*). Next, the samples were evenly distributed into cups (160 mL) and incubated at 43 °C until the pH dropped to 4.5. Cow yogurts were made with the same formulation as the reference. Three trials of yogurts were produced on three different days. All the yogurt samples were stored at 4 °C for further analysis.

#### 2.2.3. Physicochemical Analysis

pH, viscosity, and titratable acidity were measured weekly for nine weeks. pH was measured by a pH meter (Apera Instruments, LLC., Columbus, OH, USA) at room temperature. The viscosity was analyzed using a Brookfield DV-I prime viscometer (Brookfield Engineering Laboratories, Inc., Middleboro, MA, USA) with no.3 spindle at 50 rpm and recorded the reading at 30 s in mPa·S. Titratable acidity was determined by titrating with 0.1 M standard sodium hydroxide. Total solids, protein, fat, and ash content were determined using standard Association of Official Analytical Chemists procedures (AOAC) [39]. The content of carbohydrates was calculated by the difference between total solids and other solid contents. All the measurements were analyzed in triplicates, and the results were expressed as mean values with standard deviations.

#### 2.2.4. Probiotic Survivability Analysis

Probiotic viability was tested weekly for nine weeks according to the procedures from Chr. Hansen [40]. Ten grams of samples were obtained from yogurts and put in 90 mL peptone dilution water. After being diluted in a series of peptone water, one mL solution from each dilution (10^−^^7^, 10^−^^8^, 10^−^^9^) was transferred to the sterile Petri dishes. BSM agar with BSM supplement was used to enumerate BB-12 at 37 °C for 72 h under anaerobic conditions. *Lactobacillus acidophilus* LA-5 was selectively grown on de Man, Rogosa and Sharpe (MRS) with clindamycin at 37 °C in an anaerobic environment for 72 h.

#### 2.2.5. Microstructure Analysis

The microstructure of the yogurt sample was analyzed by using JSM 6060 Scanning Electron Microscope (SEM) (JEOL USA, Inc., Peabody, MA, USA) according to the method previously mentioned by Walsh et al. [34] with slight modifications. Yogurt samples were prepared in the agarose disks. Sections of yogurt samples were fractured with a blade and fixed in Karnovsky’s solution overnight. The next day, the samples were rinsed in cacodylate buffer (pH 7.2) and stored in cacodylate buffer overnight. Then, the samples were dehydrated in a different concentration of ethanol to remove extra water. Next, they were snap-frozen in liquid nitrogen and followed by critical point drying with liquid carbon dioxide. The fragments were mounted on aluminum SEM stubs and coated with gold and palladium by vacuum evaporation. The SEM was imaged at 20 kV (×5500 magnification).

#### 2.2.6. Sensory Evaluation Analysis

The sensory evaluation study was approved by the University of Vermont Institutional Review Board (STUDY00002058). Informed consent was obtained from all subjects involved in the study. Participants were recruited from the University of Vermont using research study flyers and then screened for age, allergies, and healthiness. Qualified participants were selected if they were above 21 years old, did not have any known food allergies, and were able to consume and taste dairy products and hemp extract containing cannabinoids. After signing off the consent form, participants (N = 19) were then trained on how to taste the samples and score the flavor attributes on the sensory evaluation form. Each participant received eight anonymous yogurt samples and one sensory evaluation form. Water and plain crackers were provided to clean the palate between each sample. The study was conducted on the campus of the University of Vermont. Descriptive analysis and check-all-that-apply (CATA) was used based on Alqahtani et al.’s protocol [41] with some modifications. Participants were asked to score the hemp extract flavor, goaty flavor, and aftertaste using a 5-point hedonic scale ranging from 1 = very weak flavor, almost undetectable to 5 = extremely intensive flavor. The consistency and overall acceptance were graded from 1 = dislike extremely to 5 = like extremely. After scoring the samples on the 5-point hedonic scale, the participants were asked to complete a CATA questionnaire to identify 5 attributes such as creamy, sweet, acid, milk/dairy flavor, and gummy.

#### 2.2.7. Statistical Analysis

All tests were carried out in three replications. A Q-Q plot and Shapiro test were used to evaluate the normality of data. Data that were not normally distributed were log-transformed. Results were analyzed using a mixed-effect model. The significance of the difference between mean values was estimated by the Tukey post hoc test at a *p* < 0.05. The standard deviation (±SD) was determined for all reported mean values. The sensory evaluation data were analyzed by the Kruskal–Wallis test and Chi-square test for the descriptive analysis and check-all-that-apply (CATA) questionnaire, expressing in medians ± SD. All the statistical analyses were performed using R version 4.2.2.

## 3. Results and Discussion

### 3.1. The Physicochemical Properties

The chemical composition of goat milk and cow milk yogurts is shown in Table 1. The yogurt composition varied depending on the milk type, manufacturing, and fortification process. As expected, goat milk yogurt had a lower fat content (*p* < 0.001) and a higher amount of ash than cow milk yogurt (*p* = 0.003). However, adding PWP and hemp extract did not change the chemical composition of both goat and cow milk yogurt. There was no difference in the total solids, protein, and carbohydrates between the goat and cow milk yogurt samples.

**Figure 2:**
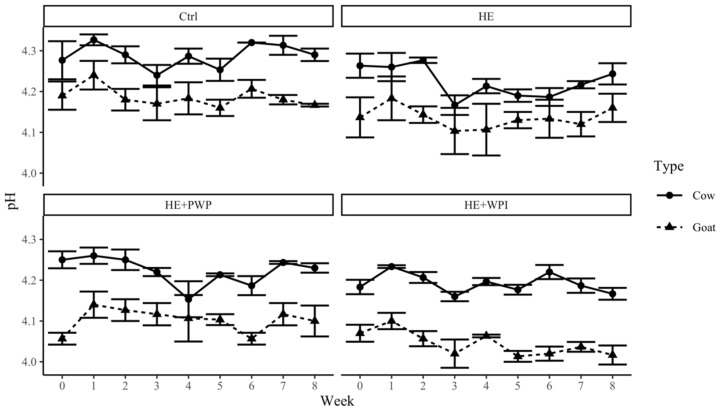
Changes in pH and titratable acidity of goat and cow milk yogurt during storage. Ctrl: control group; HE: hemp extract; PWP: polymerized whey protein isolate; WPI: unheated whey protein isolate.

**Figure 3:**
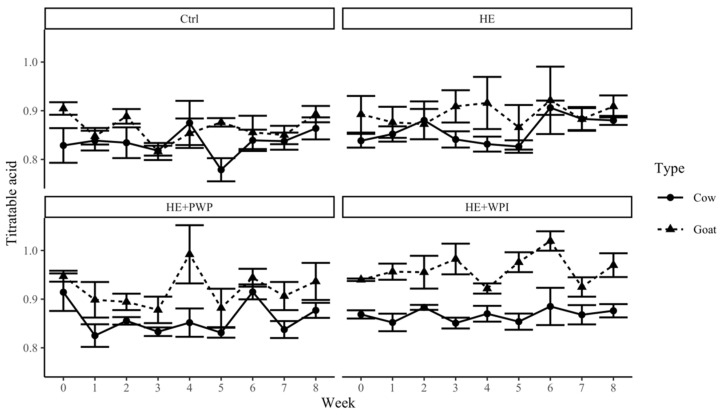
Changes in titratable acidity of goat and cow milk yogurt during storage. Ctrl: control group; HE: hemp extract; PWP: polymerized whey protein isolate; WPI: unheated whey protein isolate.

The changes in pH (Figure 2) and titratable acidity (Figure 3) in goat milk yogurt were relatively stable during the shelf-life test in each group. These results were different from other studies which showed a gradual pH decrease in goat milk and cow milk yogurts during storage. The possible explanation might be that the initial pH of the yogurt sample was lower than 4.5, and the changes during storage were insignificant. There were no significant differences in pH among the control, HE, and HE+PWP goat milk yogurt groups, whereas a significantly lower pH was found in the HE+WPI goat milk yogurt group (*p* = 0.0168). In cow milk yogurt, control cow yogurt had a higher pH than other groups (*p* = 0.0061). These differences in pH may be associated with the addition of HE, the nature of whey protein (native states or polymerized), or the proteolysis of whey protein by lactic acid [42,43,44]. Studies showed the pH varied differently in yogurt with WPI. Fang and Guo’s study [45] showed that yogurt with unheated liquid whey protein had a lower pH than the control and the ones with heated whey protein solution. While other studies indicated that there was no difference in pH in yogurt after the addition of WPI or PWP [32,44].

There was no difference in titratable acidity among the four groups of goat and cow milk yogurts. The pH of the goat milk yogurts was 0.1–0.2 units lower than cow milk yogurts, and correspondingly, the titratable acidity of goat milk yogurt was higher than that of cow milk yogurt. This result conflicted with previous studies that showed cow milk yogurt was characterized by a higher titratable acidity than goat milk yogurt [46,47]. The difference between goat and cow milk yogurt was associated with the type of milk, the milk quality, and starter cultures. The chemical composition and buffer capacity varied between different types of milk, which might influence the fermentation kinetic and lactic acid production [32,48,49].

The viscosity of goat milk yogurt was lower than cow milk yogurt in each group (*p* < 0.0001). The viscosity of control goat milk yogurt and control cow milk yogurt was 25.76 ± 14.07 mPa·S and 57.58 ± 24.88 mPa·S, respectively. Previous studies reported that goat milk has a lower content of casein, especially α_S1_-casein, resulting in poorer gelation properties and lower apparent viscosity in goat milk yogurt than in cow milk yogurt [15]. Compared with the control, the addition of HE did not affect the viscosity. After adding PWP, the viscosity of goat milk yogurt increased to 88.11 ± 29.57 mPa·S from 25.76 ± 14.07 mPa·S in the control group (*p* = 0.002). Adding WPI also increased the viscosity to 57.48 ± 32.99 mPa·S in goat milk yogurt, but it was not comparable with the increase by PWP. This difference was probably due to the formation of aggregates between PWP and casein micelles via hydrophobic interactions, while the unpolymerized whey protein, WPI, increased the viscosity by changing the casein/whey protein ratio in goat milk [50,51]. Wang et al. [31] indicated that native whey protein exhibits much lower viscosity than PWP due to their small molecule weight and approximate spherical shapes. In the heating process, whey protein polymers were formed and aggregated with increased molecule weight. Therefore, yogurts with PWP had a higher viscosity than the ones with WPI.

### 3.2. Probiotic Survivability

**Figure 4:**
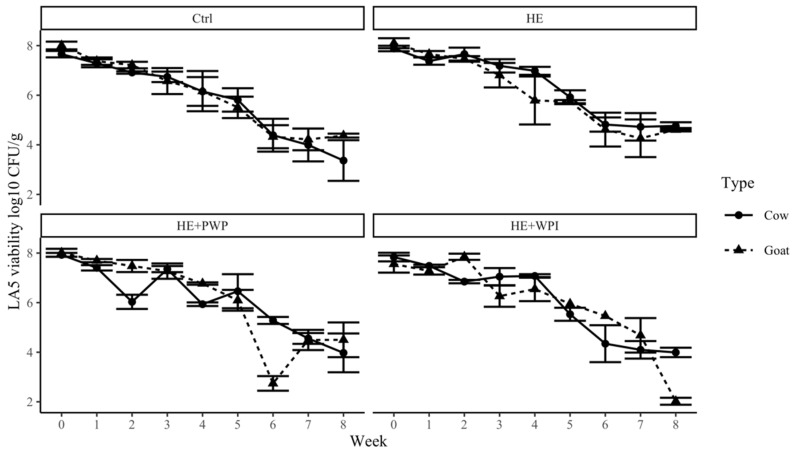
Survivability of *Lactobacillus acidophilus* LA-5 in both goat and cow milk yogurt during storage. Ctrl: control group; HE: hemp extract; PWP: polymerized whey protein isolate; WPI: unheated whey protein isolate.

To elicit pronounced health benefits, it is essential to maintain a high number of viable probiotics in yogurts during manufacturing and shelf life. The addition of HE, WPI, and PWP did not significantly change the number of probiotics. However, the probiotics showed different behavior in goat and milk yogurt samples. The initial populations of *Lactobacillus acidophilus* LA-5 and *Bifidobacterium* BB-12 in fresh cow and goat milk yogurt were about 10^8^ cfu/g. *Lactobacillus acidophilus* LA-5 was above 10^6^ cfu/g in the first 5 weeks; then, it quickly dropped below 10^4^ cfu/g (Figure 4). Tian et al. [52] also reported a similar trend of *Lactobacillus acidophilus* LA-5 survivability during storage. The presence of *S. thermophilus* in the starter cultures creates an anaerobic environment as an oxygen scavenger, which might benefit the growth and survival of *Lactobacillus acidophilus* LA-5 in yogurts [53].

**Figure 5:**
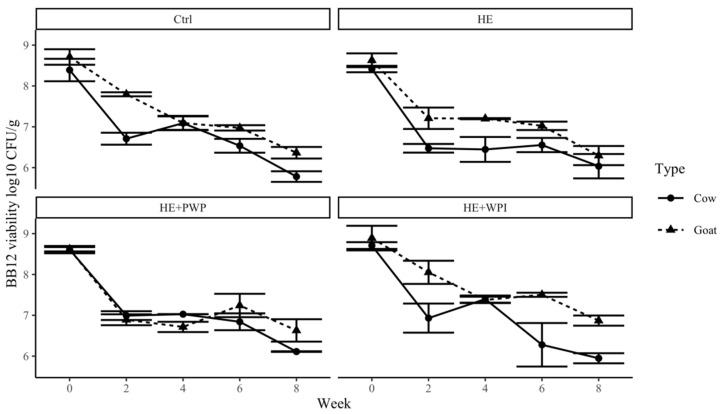
Survivability of *Bifidobacterium* BB-12 in both goat and cow milk yogurt during storage. Ctrl: control group; HE: hemp extract; PWP: polymerized whey protein isolate; WPI: unheated whey protein isolate.

The viability of BB-12 showed a slight decrease in the first two weeks but remained above 10^6^ cfu/g at the end of the ninth week (Figure 5). Generally, BB-12 showed better survivability than LA-5 during the storage of yogurt samples. Similar results were also reported in previous studies [31,47,52]. Our study noted that the BB-12 in goat milk yogurt maintained higher viability than in cow yogurt in the nine-week storage. Some researchers have reported that goat milk might be a better carrier for *Lactobacilli* and *Bifidobacterium* growth than cow milk [53]. They indicated that goat milk contains higher amounts of minerals than cow milk, which is also shown in our study in Table 1. Some minerals (e.g., calcium, zinc, and magnesium) may influence the growth of lactic acid bacteria since they are an essential component of enzymatic complexes involved in lactose fermentation [53,54]. However, the results were inconsistent because the milk composition, ingredients, and methods varied in yogurt production. Therefore, further investigation is needed to compare probiotic survivability in goat and cow milk yogurt.

### 3.3. Microstructure

**Figure 6:**
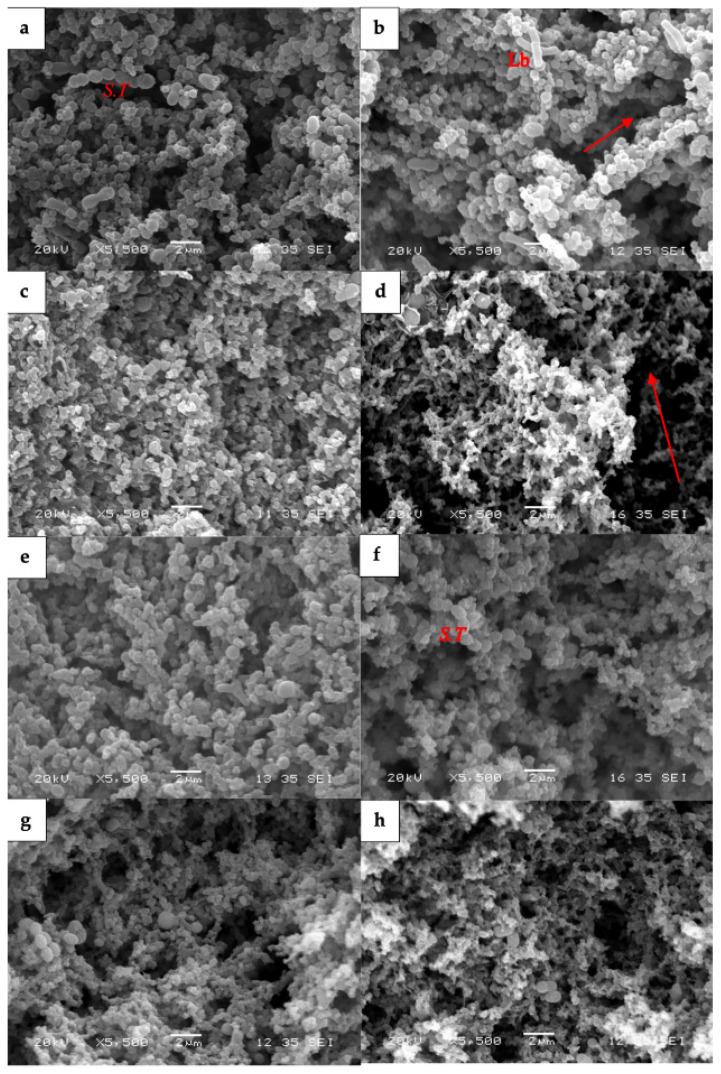
Scanning electron microscopy (SEM) photographs of goat milk yogurts and cow milk yogurt: (**a**) goat milk yogurt control, (**b**) goat milk yogurt with HE, (**c**) goat milk yogurt with HE and WPI, (**d**) goat milk yogurt with HE and PWP, (**e**) cow milk yogurt control, (**f**) cow milk yogurt with HE, (**g**) cow milk yogurt with HE and WPI, (**h**) cow milk yogurt with HE and PWP. S.T: *Streptococcus thermophilus*; Lb: *Lactobacillus*; arrow in red: space voids.

Scanning electron microscopy (SEM) images (Figure 6) illustrated the differences in the microstructure of the yogurts, such as particles and pore sizes, which provided a deeper insight into textural properties. Lactic acid bacteria were easily observed and buried in all the micrographs, indicating abundant probiotics in the yogurt samples. The addition of HE in goat milk yogurt did not significantly change the microstructure in SEM images. The micrographs (Figure 6a,b) of the control and HE goat milk yogurt consisted of relatively large particles interspaced by voids that presented initially with entrapped water and fat. The void spaces were larger and unevenly distributed in the protein matrix. The addition of WPI into HE-infused goat yogurts (Figure 6c) exhibited larger aggregates and clusters, and the structure became more rigid compared with the control and HE-infused goat milk yogurts (Figure 6a,b). The goat milk yogurt with PWP (Figure 6d) showed a more organized network structure, indicating better water immobilization with other components. Also, the casein protein matrix was less defined than other groups. A similar phenomenon was also observed in cow milk yogurt samples with PWP (Figure 6h).

Similarly, there was no significant difference between the control and HE cow milk yogurt (Figure 6e,f). After adding WPI, the cow milk yogurt had a denser matrix with easily identified space voids and particles (Figure 6g). Similar results were also reported by previous studies, which showed larger particles in cow milk yogurt with WPI (3%) compared to yogurt without WPI [44,55]. The changes in the ratio of casein and whey protein created a more robust structure during storage. In contrast, conflicting findings from two papers indicated no significant changes of complexes between the casein and whey protein in yogurt structure, which might be due to the different formulas and the methods of yogurt production [56,57]. The addition of PWP (Figure 6h) in cow milk yogurt showed fine protein complexes and gel networking, reflecting better gelation properties than cow milk yogurt with WPI.

These differences between the control and PWP yogurt groups may be attributed to the gelation property of the PWP [33]. Casein was a major protein in milk, and its composition greatly affected the protein coagulation during the yogurt fermentation. Compared with cow milk, goat milk has a higher non-nitrogen protein, and a lower casein content, especially a lower concentration of α_s1_-casein [48]. α_s1_-casein is the main protein holding the micelles together at cold temperatures, leading to differences in the structure of the protein network of each yogurt type [49]. Therefore, goat milk yogurt had a weaker gel structure and lower consistency than cow milk yogurt due to the lower content of α_s1_-casein. After adding PWP, the casein-PWP complexes were formed mainly through hydrophobic interactions, which could improve the gel structure of both goat and cow milk yogurt. Compared with unheated whey protein (WPI), the functional protein, β-lactoglobulin, in PWP was pre-heated and partially unfolded to form aggregates. Then, the free cysteines (-SH) and hydrophobic sites were exposed. Hydrophobic interactions occurred between the β-lactoglobulin or whey protein aggregates and casein micelles when the pH was lowered to the isoelectric point of β-lactoglobulin in the fermentation process. In addition, the electrostatic repulsion of PWP tended to decrease and consequently trapped water and other components. This process, also known as acid-induced cold-set gelation [58], is a reasonable explanation for the effect of PWP on improving the structure of goat milk yogurt.

### 3.4. Sensory Evaluation 

**Figure 7:**
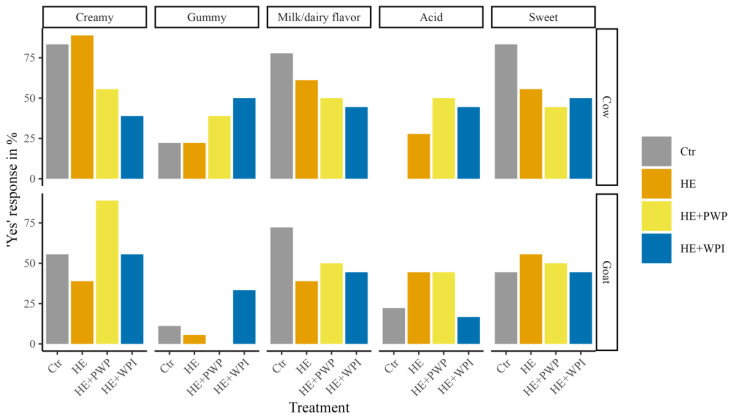
The percentage of “Yes” responses for the attributes in both cow and goat milk yogurt from the CATA test. Ctr: control group; HE: hemp extract; PWP: polymerized whey protein isolate; WPI: unheated whey protein isolate.

The results of the sensory evaluation of goat milk yogurt are shown in Table 2. The control goat milk yogurt (G1) had the highest goaty flavor score (2.50 ± 1.64). When HE, WPI, and PWP were added to goat milk, the scores of goaty flavor decreased to 1.00 ± 1.02, 1.00 ± 0.88, and 1.00 ± 0.98, respectively. Goat milk with HE (GH) showed the most distinguished hempy flavor (4.00 ± 1.40) and the lowest overall acceptance score (2.00 ± 1.34). Although the hempy flavor and overall acceptance were not significantly improved with the addition of PWP and WPI in goat milk yogurt, their consistency showed a great improvement compared to the GH group (*p* = 0.003). For HE-infused goat milk yogurt treated with PWP, panelists reported the least hempy flavor and the highest consistency. However, there was no statistical difference in the sensory attributes between PWP and WPI in goat milk yogurt, although goat milk yogurt with PWP had better consistency and aftertaste. Similarly, in cow milk yogurt, hemp extract significantly increased the hempy flavor and decreased the overall acceptance. Compared with the effect of PWP or WPI on goat milk yogurt, PWP and WPI had no significant impact on improving consistency in cow milk yogurt. The overall acceptance of control cow milk yogurt (C1, 4.25 ± 0.86) was higher than control goat milk yogurt (G1, 3.00 ± 1.39). Surprisingly, even though the overall acceptance of cow milk yogurt with the addition of hemp extract (CH) was lower than the control cow milk yogurt (C1) (*p* = 0.008), it was more acceptable than the WPI groups (*p* = 0.030) despite the similar scores of other attributes. From the responses of the check-all-that-applied (CATA) test (Figure 7), goat milk yogurt with PWP had better performance on creamy and gummy attributes. Interestingly, no response of “gummy” was reported in PWP-fortified goat milk yogurt samples, while most people indicated “gummy” in WPI groups. Cow milk yogurt samples with PWP also exhibited less gummy and more creamy attributes than the samples with WPI.

Although the control goat milk yogurt had similar protein and total solids content to the control cow milk yogurt (Table 1), the goat milk yogurt showed a lower consistency and softer gel than cow milk yogurt. With the addition of WPI and PWP, the consistency of goat milk yogurt was considerably improved to 3.00 ± 1.31, 4.00 ± 0.94 from 2.00 ± 1.24 in the control (Table 2). These results indicated that the consistency of goat milk yogurt was affected by the physicochemical properties of the milk proteins, especially the lower content of α_s1_-casein. In addition, the findings also echoed the SEM results that the microstructure of goat milk yogurt was improved after adding PWP. Similar behaviors were also observed in several studies [31,32,33,59]. The optimized gel structure and consistency after adding PWP because of the hydrophobic bonds emphasized the importance of PWP on the microstructural and sensory properties of goat milk yogurt.

The popularity and consumer acceptance of goat milk yogurt were closely related to its consistency and off-flavor. Some studies showed that the fermentation process, especially with the probiotic bacteria, can help improve the sensory properties of goat milk yogurts [60,61]. The increased acetaldehyde content in the fermentation was responsible for the typical milky-fermentative taste and covered the caprine smell and taste. For goat milk yogurts with PWP and WPI, adding whey protein to milk increased the content of amino acids (e.g., threonine and valine amino acids) which were precursors in the acetaldehyde formation pathway. In addition, Wang et al. [62] reported that PWP could mitigate the goaty flavor by trapping the short/medium-chain fatty acids in goat milk yogurt, especially capric acid. The binding affinity of PWP to hemp terpenes may explain the lack of evidence for hempy flavor mitigation, and very few studies explored the flavor reduction of terpenes by PWP. The hydrophobic pockets in β-lactoglobulin and/or whey protein aggregates are the primary binding sites for the hempy flavor compounds, which may also bind fatty acids [63]. It is possible that different flavor compounds competed for the same binding sites; however, the mechanism is not clear, and more studies are needed in the future. Our other study observed that the combination of PWP and β-cyclodextrin had better performance on hempy flavor mitigation in HE-infused ice cream [64], which indicated that the flavor mitigation effect of PWP could be improved with the addition of other ingredients, like β-cyclodextrin. Na et al. [65] reported that the odor intensity of the encapsulated fish oil decreased by 30% of its original score with the combination of whey protein concentration and cyclodextrin. Later, results from Wang et al. [62] showed that the combination of PWP and β-cyclodextrin had better texture and consistency in fermented goat milk than the effect of individual compounds. On the other hand, the involvement of a larger panelist size and longer training time may increase the power for detecting the difference between treatment groups, especially for the mitigating effect of PWP on hemp flavor in goat milk yogurts (*p* = 0.070); therefore, future studies need to include more participants and training sections.

## 4. Conclusions

Goat milk yogurt infused with hemp extract is an excellent carrier for delivering probiotics. The survival rates of *Bifidobacterium* BB-12 remained above 10^6^ cfu/g over the 9 weeks of storage. *Lactobacillus acidophilus* LA-5 maintained viable counts of about 10^6^ cfu/g during the first five weeks and then decreased sharply. HE-infused goat milk yogurt with PWP presented a more desired microstructure and consistency by forming a compact gel networking. PWP could also reduce goaty and hempy flavor, likely due to the hydrophobic interactions between PWP, short-chain fatty acids, and hemp terpenes. In short, PWP can improve the microstructural and sensory properties of hemp extract-infused goat milk yogurt.

## 5. Patents

A patent (#WO 2023235740) has been filed and approved for the methods in this paper.

## Figures and Tables

**Table 1:** Chemical composition of goat milk and cow milk yogurts.

Item (%)	Control		HE		HE+WPI		HE+PWP	
	GY	CY	GY	CY	GY	CY	GY	CY
Total solid	18.23 ± 1.11	18.04 ± 1.94	17.78 ± 1.92	19.57 ± 0.37	17.60 ± 2.10	17.06 ± 2.13	19.22 ± 1.36	19.56 ± 0.37
Fat	2.90 ± 0.18 **	3.40 ± 0.25	2.92 ± 0.40 **	3.39 ± 0.15	2.68 ± 0.20 **	3.21 ± 0.19	2.84 ± 0.10 **	3.49 ± 0.14
Protein	3.64 ± 0.06	3.23 ± 0.42	3.44 ± 0.43	3.45 ± 0.21	3.53 ± 0.45	3.79 ± 0.11	3.05 ± 0.11	4.10 ± 0.51
CHO	10.81 ± 0.97	10.71 ± 1.83	10.68 ± 2.09	12.05 ± 0.16	10.52 ± 2.85	10.32 ± 2.23	12.55 ± 1.12	11.30 ± 0.31
Ash	0.87 ± 0.07 *	0.70 ± 0.03	0.72 ± 0.09 *	0.67 ± 0.02	0.87 ± 0.16 *	0.65 ± 0.00	0.77 ± 0.12 *	0.66 ± 0.02

All the values in this table were expressed as means ± standard derivations. GY: goat milk yogurt; CY: cow milk yogurt; HE: hemp extract; WPI: unheated whey protein isolate; PWP: polymerized whey protein isolate. CHO: carbohydrates. * *p* < 0.05, ** *p* < 0.001 compared with the corresponding CY, Tukey post hoc test.

**Table 2:** Sensory evaluation scores of goat milk (G) and cow milk (C) yogurts.

Yogurts	Attributes				
	Hempy Flavor	Goaty Flavor	Consistency	Aftertaste	Overall Acceptance
C1	1.00 ± 0.54 ^a^	1.00 ± 1.34 ^a^	4.00 ± 1.05 ^a^	2.00 ± 1.64 ^a^	4.25 ± 0.86 ^a^
CH	3.00 ± 1.32 ^b^	1.00 ± 0.88 ^a^	4.00 ± 0.96 ^a^	3.25 ± 1.20 ^a^	3.25 ± 1.27 ^b^
CWPI	3.00 ± 1.41 ^b^	1.00 ± 0.71 ^a^	3.00 ± 1.86 ^a^	3.00 ± 1.26 ^a^	2.00 ± 1.28 ^c^
CPWP	3.00 ± 1.23 ^b^	1.00 ± 0.97 ^a^	3.00 ± 1.59 ^a^	3.00 ± 1.26 ^a^	3.00 ± 1.15 ^bc^
G1	1.00 ± 0.64 ^A^	2.50 ± 1.64 ^A^	2.00 ± 1.24 ^A^	3.00 ± 1.20 ^A^	3.00 ± 1.39 ^A^
GH	4.00 ± 1.40 ^B^	1.00 ± 1.02 ^A^	2.00 ± 1.17 ^A^	3.00 ± 1.23 ^A^	2.00 ± 1.34 ^A^
GWPI	2.75 ± 1.38 ^B^	1.00 ± 0.88 ^A^	3.00 ± 1.31 ^B^	2.00 ± 1.08 ^A^	3.00 ± 1.00 ^A^
GPWP	2.50 ± 1.22 ^B^	1.00 ± 0.98 ^A^	4.00 ± 0.94 ^B^	3.00 ± 0.90 ^A^	3.00 ± 1.16 ^A^

All the values were expressed as medians ± standard derivations. Medians in the same column with different subscripts (a, b, c) were significantly different among cow milk yogurts; medians in the same column with different subscripts (A or B) were significantly different among goat milk yogurts. *p* < 0.05 compared to the same column based on Kruskal–Wallis test. C1, G1 = cow (C), goat milk (G) yogurt control. CH, GH = cow, goat milk yogurt infused with hemp extract. CPWP, GPWP = hemp extract infused cow, goat milk yogurt with PWP. CWPI, GWPI = hemp extract infused cow, goat milk yogurt with WPI.

## Data Availability

The original contributions presented in this study are included in the article. Further inquiries can be directed to the corresponding author.
